# Victor A. McKusick, the “Father of Medical Genetics”

**DOI:** 10.3390/audiolres11040058

**Published:** 2021-11-25

**Authors:** Alessandro Martini, Matteo Cassina

**Affiliations:** 1 Padova University Research Center “International Auditory Processing Project in Venice (I-APPROVE)”, “Santi Giovanni e Paolo” Hospital, 30122 Venice, Italy; 2 Clinical Genetics Unit, Department of Women’s and Children’s Health, University of Padova, 35128 Padova, Italy

The Special Issue “Genetics of hearing loss” is dedicated to Victor A. McKusick, on the 100th anniversary of his birth.

McKusick (1921–2008) is widely known as the “father of medical genetics” and editor of Mendelian Inheritance in Man (MIM). According to Francis Collins (NIH Bethesda) “*McKusick was the driving force for moving genetics beyond the tidy realm of flies and mice in the research lab into the messier realm of the medical clinic*”. “*No description of McKusick’s impact on science is complete without mentioning his prescient call for mapping the human genome. In August 1969, at the International Conference on Birth Defects in The Hague, McKusick proposed that mapping all human genes would be useful for understanding basic derangements in birth defects*” [1]. The first edition of his classic “Mendelian Inheritance in Man: Catalogs of autosomal dominant, autosomal recessive, and X-linked phenotypes” was published in 1966; eleven updated editions were published between 1969 and 1998 [2,3] and followed by an online edition (Online Mendelian Inheritance in Man-OMIM) [4]. The content of OMIM is derived exclusively from the published biomedical literature and is updated daily. Currently, OMIM is authored and edited at the McKusick-Nathans Institute of Genetic Medicine, Johns Hopkins University School of Medicine, under the direction of Dr. Ada Hamosh. It now contains 26,058 full-text overviews describing phenotypes and genes; among these, 6188 entries are descriptions of phenotypes with a known molecular basis, and 16,561 entries are gene descriptions (www.omim.org/statistics/entry, accessed on 17 September 2021).

McKusick attended Tufts University (1940–1943), then moved to Johns Hopkins University School of Medicine (M.D., 1946) in Baltimore and trained as a cardiologist, particularly interested in the study of heart sounds and murmurs. However, during his cardiology practice, the encounter with a patient affected by Marfan syndrome triggered McKusick’s switch to medical genetics and to the study of this disease and other inherited disorders of connective tissue [5,6,7,8,9]. In 1957, he founded the first medical genetics clinic at Johns Hopkins, serving as its director until 1975; moreover, he chaired the department of medicine at Johns Hopkins (1973–1985), where he remained as a professor of medical genetics.

McKusick was co-founder of the European School of Genetic Medicine (www.eurogene.org, accessed on 17 September 2021) and had a special relationship with Italy, the Country where the courses in medical genetics took place (Figure 1 and Figure 2). In McKusick’s obituary, Giovanni Romeo (professor of medical genetics at the University of Bologna) wrote “*Victor was a living legend both at Johns Hopkins Hospital where he spent all his professional life, starting as a medical student in 1943, and all over the world. His presence on the international scene of modern Medical Genetics has been so prominent (from the launching of the 1st Birth Defects Conference to the organization of the Human Gene Mapping conferences together with Frank Ruddle and to the foundation of HUGO-Human Genome Organization) and his scientific achievements have been so numerous that I will have to concentrate only on his links with Europe using many personal memories arising from the courses of the European School of Genetic Medicine. He founded the School in 1988 in collaboration with many European medical geneticists who were to become the leaders of the newly reorganized European Society of Human Genetics a few years later (in 1990)*” [10].

McKusick published more than 800 scientific papers and books and he gave a great contribution also to the study of the genetics of deafness [11,12,13,14,15], especially in collaboration with Bruce W. Konigsmark. McKusick’s “Mendelian Inheritance in Man” [2,3] and Konigsmark’s “Genetic and Metabolic Deafness” [16] were long considered the “bibles” by clinicians involved in the management of patients with hereditary hearing loss.

## Figures and Tables

**Figure 1:**
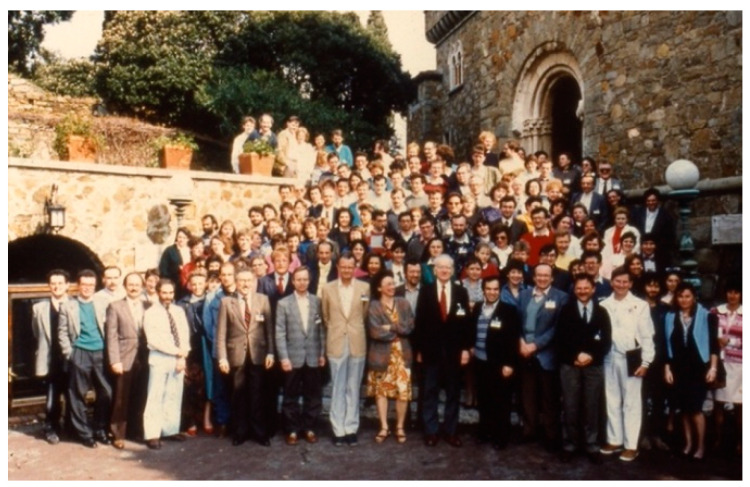
European School of Genetic Medicine, first course in medical genetics-Sestri Levante, Italy (1988) (courtesy of Prof. Giovanni Romeo).

**Figure 2:**
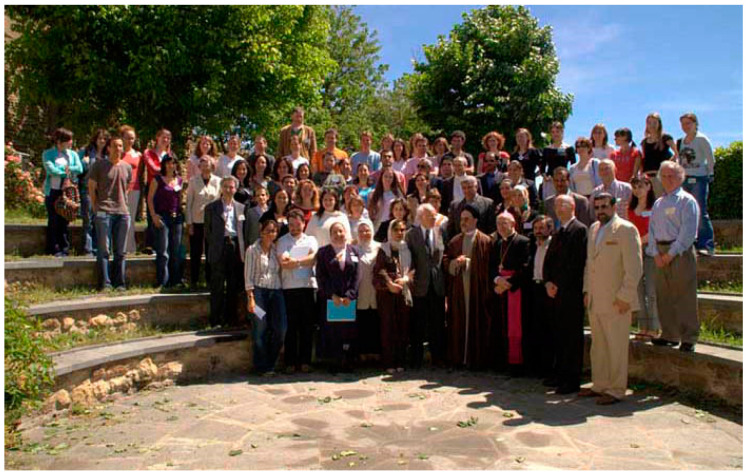
European School of Genetic Medicine, 20th course in medical genetics-Bertinoro, Italy (2007) (courtesy of Prof. Giovanni Romeo).

## References

[1] Collins F.S. (2008). Retrospective: Victor A. McKusick (1921–2008). Science.

[2] McKusick V.A. (1966–1992). Mendelian Inheritance in Man: Catalogs of Autosomal Dominant, Autosomal Recessive, and X-Linked Phenotypes.

[3] McKusick V.A. (1994–1998). Mendelian Inheritance in Man: A Catalog of Human Genes and Genetic Disorders.

[4] Online Mendelian Inheritance in Man, OMIM® McKusick-Nathans Institute of Genetic Medicine.

[5] McKusick V.A. (1955). The cardiovascular aspects of Marfan’s syndrome: A heritable disorder of connective tissue. Circulation.

[6] McKusick V.A. (1955). Heritable disorders of connective tissue. I. The clinical behavior of hereditary syndromes. J. Chronic Dis..

[7] McKusick V.A. (1955). Heritable disorders of connective tissue. II. The biology of normal connective tissue. J. Chronic Dis..

[8] McKusick V.A. (1955). Heritable disorders of connective tissue. III. The Marfan syndrome. J. Chronic Dis..

[9] McKusick V.A. (1957). The genetic behaviour of heritable disorders of connective tissue. Acta Genet. Stat. Med..

[10] Romeo G. (2008). Victor McKusick, 1921–2008: The founder of medical genetics as we know it. Eur. J. Hum. Genet..

[11] Konigsmark B.W., McKusick V.A. (1966). Hereditary deafness. Volta Rev..

[12] Mengel M.C., Konigsmark B.W., Berlin C.I., McKusick V.A. (1967). Recessive early-onset neural deafness. Acta Otolaryngol..

[13] Eldridge R., Berlin C.I., Money J.W., McKusick V.A. (1968). Cochlear deafness, myopia, and intellectual impairment in an Amish family. Acta Otolaryngol..

[14] Mengel M.C., Konigsmark B.W., Berlin C.I., McKusick V.A. (1969). Conductive hearing loss and malformed low-set ears, as a possible recessive syndrome. J. Med. Genet..

[15] Mengel M.C., Konigsmark B.W., McKusick V.A. (1969). Two types of congenital recessive deafness. Eye Ear Nose Throat Mon..

[16] Konigsmark B.W., Gorlin R.J. (1976). Genetic and Metabolic Deafness.

