# Correction: Martini, A.; Cassina, M. Victor A. McKusick, the “Father of Medical Genetics”. *Audiol. Res.* 2021, *11*, 636–638

**DOI:** 10.3390/audiolres12010011

**Published:** 2022-02-17

**Authors:** Alessandro Martini, Matteo Cassina

**Affiliations:** 1 Padova University Research Center “International Auditory Processing Project in Venice (I-APPROVE)”, “Santi Giovanni e Paolo” Hospital, 30122 Venice, Italy; 2 Clinical Genetics Unit, Department of Women’s and Children’s Health, University of Padova, 35128 Padova, Italy

In the original article [1] there was a mistake in the published version of Figure 3. The photo showed Prof. Mario Capecchi (on the right) and not Prof. Giovanni Romeo. Figure 3, its legend and its reference in the manuscript have been deleted. The authors apologize for any inconvenience caused. The original article has been updated.
